# The bacteria inside human cancer cells: Mainly as cancer promoters

**DOI:** 10.3389/fonc.2022.897330

**Published:** 2022-08-12

**Authors:** Wei Zhu, Jing-Zi Wang, Zhixian Liu, Ji-Fu Wei

**Affiliations:** ^1^ Jiangsu Cancer Hospital, Jiangsu Institute of Cancer Research, The Affiliated Cancer Hospital of Nanjing Medical University, Nanjing, China; ^2^ Research Division of Clinical Pharmacology, the First Affiliated Hospital of Nanjing Medical University, Nanjing, China; ^3^ Department of Urology, Children’s Hospital of Nanjing Medical University, Nanjing, China

**Keywords:** bacteria, cancer cells, *Fusobacterium nucleatum* (*F.n*), cancer treatment, colorectal cancer

## Abstract

The roles of the microbiome in human beings have become clearer with the development of next-generation sequencing techniques. Several pieces of evidence showed strong correlations between the microbiome and human health and disease, such as metabolic disorders, infectious diseases, digestive system diseases, and cancers. Among these diverse microbiomes, the role of bacteria in human cancers, especially in cancer cells, has received extensive attention. Latest studies found that bacteria widely existed in cancers, mainly in cancer cells and immune cells. In this review, we summarize the latest advances in understanding the role of bacteria in human cancer cells. We also discuss how bacteria are transported into cancer cells and their physiological significance in cancer progression. Finally, we present the prospect of bacterial therapy in cancer treatment.

## Introduction

Human microbiome is composed of bacteria, viruses, eukaryotic fungi, and protozoa (1). The roles of the microbiome in humans have become clearer with the development of next-generation sequencing techniques (2, 3). In healthy conditions, the human host and microbiome are symbiotic. The human host can provide a nutritious microenvironment for microbiome to help the human host with the metabolism and digestion process (4, 5). Several pieces of evidence showed strong correlations between the microbiome and human disease, such as metabolic disorders (6), infectious diseases (7), digestive system diseases (8), and cancers (9). Many microorganisms were related to the development of human cancers, such as bacteria and viruses, including human papillomavirus (HPV), hepatitis B virus (HBV), hepatitis C virus (HCV), Epstein–Barr virus (EBV) (9), and human endogenous retroviruses K (HERV-K) (10). There are three trillion bacterial members in human bodies, which can regulate the comprehensive interplay of physiological processes and disease susceptibilities (11). The high genetic diversity of bacteria encodes excellent mechanistic and metabolic competences that not only influence their own microbial niche but also regulate host tissue-specific and immune cell functions (12). Bacteria may also have health benefits, such as producing metabolites to fight cancers (13, 14). Butyrate, which is metabolized by intestinal bacteria, provides energy sources for colonocytes and suppresses inflammation and carcinogenesis by affecting immunity, gene expression, and epigenetic modification (14). Some bacteria, such as *Lactococcus*, Clostridia, *Shigella*, Bifidobacteria, *Listeria*, *Vibrio*, *Salmonella*, and *Escherichia*, have demonstrated significant potential for invasion and colonization in tumors sites, thereby leading to tumor clearance and retardation of neoplasm growth. Moreover, some bacteria such as Clostridia strains and *Bifidobacterium longum* are able to survive and colonize in the hypoxic condition of the tumor to destroy it (15, 16).

Recently, the role of bacteria in cancer development has drawn more attention (17). A well-established link between bacteria and human cancer is *Helicobacter pylori* and gastric cancer (18). The cytotoxin-associated gene A (CagA) produced by *H. pylori* is one of the first bacterial proteins identified to promote human cancer. CagA could activate the oncogenic signal transduction pathways, interfere with cell cycle and cell death, and increase the risk of gastric cancer (19, 20). Currently, only 11 organisms have been formally linked as potential causes of human cancers, including *H. pylori* (21), the only bacterium recognized by the International Agency for Research on Cancer (IARC). Despite the recent advances in microbiological and microbiome research, the protumorigenic microbe list by IARC has not been updated for more than one decade. Recent studies suggested that dozens of bacteria could modulate or contribute to cancers other than *H. pylori* (22), as shown in **Table 1**. Mechanically, bacteria can cause malignant tumors through the deleterious alterations in the physiological processes of the host, such as 1) chronic inflammation (38, 39), 2) antigen-driven lymph proliferation (40), 3) induction of hormones that increase the proliferation of epithelial cells (19, 20), 4) directly affecting oncogenesis through changing the cell transformation (41), or 5) interrupts the cellular signal by the production of carcinogenic metabolites or toxic substances, therefore, interfering with the regulation of cell growth (14, 42).

**Table 1:** Cancer-associated bacteria.

Bacteria	Cancer	Expression	Mechanism	Function	References
*Helicobacter pylori*	Gastric cancer	High	Wnt/β-catenin pathway	Regulating cellular turnover and apoptosis	(23)
			Correa pathway	Chronic inflammatory response	(24, 25)
*Fusobacterium nucleatum*	CRC	High	Invasion of CRC cells	Influencing CRC development	(26)
	OSCC	High	Invasion of OSCC cells	Pro-inflammatory cascades	(27)
*Escherichia coli*	CRC	Imbalance	Inducing inflammation, oxidative stress, changes in the cellular niche, interference and manipulation of the host cell cycle	Promoting cancer formation	(28, 29)
*Bacteroides fragilis*	CRC	High	Inducing chronic intestinal inflammation and tissue damage	Promoting colon tumorigenesis	(30–33)
			Activation of Wnt/β-catenin, NFκB pathway, and Th17 adaptive immunity	Promoting colon tumorigenesis	(30, 34–36)
*Salmonella enterica*	Gallbladder cancer	High	Activation of MAPK and AKT pathways	Cancer tumorigenesis	(37)

CRC, colorectal cancer; OSCC, oral squamous cell carcinoma; MAPK, mitogen-activated protein kinase.

Bacteria were first discovered in human cancers about 100 years ago (43). All of the above studies described the role of extracellular bacteria in human cancers. The characterization of bacteria in human cancers has not been well studied due to their low biological expression. A recent report comprehensively analyzed the microorganisms in seven human cancers and demonstrated that the bacteria in cancers are primarily located in cells, including cancer cells and immune cells (44). Moreover, Livyatan et al. (45) also verified that bacteria were alive in cancer cells, but not bacterial components.

In this review, we summarize the latest progress in understanding the role of bacteria in human cancer cells and discuss how bacteria are transported into cancer cells and their physiological significance in cancer progression. Finally, we present the prospect of bacterial therapy in tumor treatment.

## Bacteria inside human cancer cells

The microbiome of human cancer and its adjacent normal tissues was analyzed in more than 1,500 samples, and a bacterial catalog of seven different cancer types was generated (44). Cancer microbiome showed distinct microbial characteristics across different cancer types and even different subtypes (44). Visualization method showed that bacteria in cancers were mainly located in cancer and immune cells, not in the extracellular compartment (44, 45). Bacterial lipopolysaccharide (LPS) and lipoteichoic acid (LTA) were detected by immunohistochemistry (IHC) to label Gram-negative and Gram-positive bacteria, respectively (46). RNA fluorescence *in situ* hybridization (FISH), with a universal probe against bacterial 16S ribosomal RNA (rRNA), was used to detect bacterial RNA in cancer tissues (47). The examination of LPS, LTA, and bacterial 16S rRNA was performed in different cell types across 459, 427, and 354 cancer cores, respectively (44). It was found that LPS and 16S rRNA were mainly located in cancer cells and immune cells. In cancer cells, bacterial 16S rRNA was detected mostly in the cytoplasm, whereas LPS was detected in both the cytoplasm and the nucleus. LTA was only detected in macrophage cells but rarely in cancer cells and immune cells (44). To further verify the presence of bacteria inside cancer cells, the bacteria were found in close proximity to the nuclear membrane in four human breast cancer tissues by correlative light and electron microscopy (CLEM) (44). The bacteria were not detected in the nucleus, indicating that the appearance of LPS nuclear localization in some cancer cells was probably the staining of cytoplasmic perinuclear bacteria (44).

While positive FISH staining of bacterial 16S rRNA confirmed the diffused signal inside cancer cells, typical bacterial rods or cocci were rarely detected. Notably, no cell wall polymer LTA was detected in cancer cells, but many Gram-positive bacteria in human cancers were detected by 16S rDNA sequencing, suggesting that bacteria in human cancer cells may alter their envelope, perhaps result in a cell wall-deficient state, such as L-forms (48). Cell wall-deficient bacteria were only found inside cells (49, 50). In breast cancer, many intracellular cell wall-deficient bacteria were indeed found (44). Bacteria in the cytoplasm of breast cancer cells were also confirmed in the study by Fu et al. (51).

## How are bacteria transported into human cancer cells?

**Figure 1:**
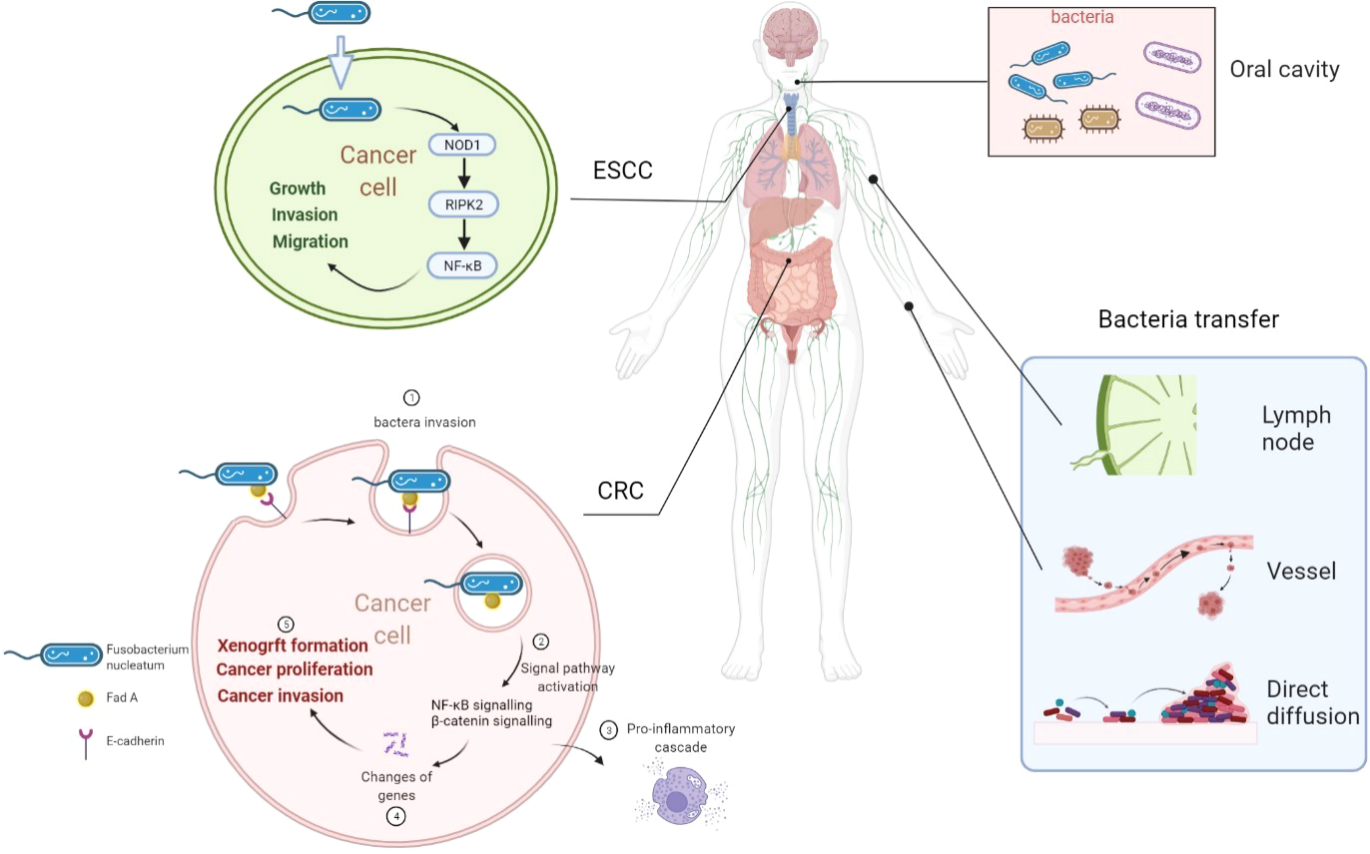
Overview of proposed mechanisms that *Fusobacterium nucleatum* invading cancer cells. *F. nucleatum* is a common bacterium in the oral cavity. *F. nucleatum* invades ESCC cells and CRC cells. *F. nucleatum* may transfer through lymph, vessel, or direct diffusion. *F. nucleatum* promotes ESCC progression by the NOD1/RIPK2/NFκB pathway. FadA is highly conserved among *F. nucleatum* and expressed on the bacterial surface. FadA binds E-cadherin on CRC cells to attach and invade CRC cells. Then, NFκB signaling and β-catenin signaling are activated to induce a pro-inflammatory cascade to promote CRC progression. ESCC, esophageal squamous cell carcinoma; CRC, colorectal cancer; *Fn*, *Fusobacterium nucleatum*; NOD1, nucleotide-binding oligomerization domain-containing protein 1; RIPK2, interacting serine threonine kinase 2; NFκB, nuclear factor-κB.

There are more than 1,000 different species totaling 1,014 microorganisms of the human intestinal microbiome. The microbiome is involved in the important normal physiological activities of the intestine, such as energetic metabolism, proliferation of epithelial cells, and resistance of pathogens (52). *Fusobacterium nucleatum* is an opportunistic commensal anaerobe in the oral cavity, implicated in various forms of periodontal diseases. Outside the oral cavity, it is one of the most prevalent species in extraoral infections. *F. nucleatum* was highly expressed in colorectal cancer (CRC) and was related to CRC development (26), indicating that an oral pathogen associated with oral squamous cell carcinoma (OSCC) might also be related to distant cancers (26, 53). Additionally, *F. nucleatum* was also associated with liver metastasis, further suggesting its role in cancer development (54). *F. nucleatum* adheres to and invades endothelial and epithelial cells *via* FadA to get systemic transmission (27, 55). This leads to the internalization of pathogens and mediated the pro-inflammatory cascade by the induction of nuclear factor (NF)-κB (NFκB) and interleukin-6, constituting a possible way by which *F. nucleatum* invades OSCC cells (55, 56). FadA is highly conserved among *F. nucleatum* and was expressed on the bacterial surface (57). It can specifically bind to the EC5 region of E-cadherin on CRC cells to attach to and invade CRC cells, which was also demonstrated in HEK293 cells (58). *F. nucleatum* is also highly expressed in esophageal squamous cell carcinoma (ESCC) tissues (59) compared with their adjacent non-tumor tissues. Recently, it was also observed by transmission electron microscopy that it has the ability to invade ESCC cells (59). But how are bacteria transported into cancer cells was not reported in detail. Combined with previous reports, we speculated that bacteria might transfer to distant cancer cells by the digestive tract (direct diffusion), lymph, or blood pathway (60). The proposed mechanisms of how bacteria invade cancer cells are shown in **Figure 1**.

## What roles do bacteria play in human cancer cells?

The study by Rubinstein et al. (58) demonstrated that FadA can bind to E-cadherin expressed on CRC cells and mediate *F. nucleatum* attachment and cell invasion. Then, FadA activates β-catenin signaling, leading to increased expression of transcription factors, oncogenes, Wnt genes, and inflammatory genes to promote CRC cell proliferation (58). Furthermore, FadA directly regulates Wnt and oncogene activation upon binding to CRC cells, but clathrin-mediated FadA internalization is also required for the inflammatory gene activation (58). In mice, CRC cells with *F. nucleatum* infection can increase their proliferation, invasive activity, and the ability to form xenograft cancers (58, 61). Another study demonstrated that *F. nucleatum* could activate toll-like receptor 4 signaling to MYD88, resulting in activation of the nuclear factor NFκB and elevated expression of miR21, which reduced the expression of the RAS GTPase RASA1 (61). In ESCC, *F. nucleatum* was associated with poor survival and promoted cancer cell growth, migration, and invasion. It can invade ESCC cells and induce the NFκB pathway through the nucleotide-binding oligomerization domain-containing protein 1 (NOD1)/receptor interacting serine threonine kinase 2 (RIPK2) pathway, leading to tumor progression. With the help of a murine spontaneous breast tumor model, it can be found that after clearing the bacteria in cancer cells, tumor weight was not affected, but lung metastasis decreased significantly. This shows that the intracellular bacteria are likely to affect the metastasis process rather than the growth of breast cancer (51). After bacteria invaded breast cancer cells, RhoA-ROCK signaling pathway can be activated to reorganize the cytoskeleton and help breast cancer cells resist the pressure from blood vessels and avoid damage during metastasis. Therefore, breast cancer cells carrying bacteria have a stronger ability to reach distant tissues for metastasis (51).

## Bacteria in cancer treatment

The usefulness of eukaryotic and prokaryotic expression systems in delivering therapeutic payloads has been explored, including cytotoxic agents (62, 63), prodrug invertase (64, 65), immunomodulators (66), tumor matrix-targeting molecules (67, 68), and siRNA (68). Prokaryotic expression systems discussed above are the most commonly used methods. They encode targeted genes depending on bacterial prokaryotic plasmids (67, 69). Eukaryotic expression systems involve the transduction of host cells, such as immune cells or tumor cells, with the eukaryotic plasmids encoding the cDNA of target genes (70).

*F. nucleatum* is highly expressed in CRC tissues. Treatment with the antibiotic metronidazole can reduce *F. nucleatum* amount, cancer cell proliferation, and cancer growth in *F. nucleatum*-positive CRC (54). Therefore, bacteria may become therapeutic targets in cancer treatment. *Mycobacterium bovis* strain bacillus Calmette–Guérin (BCG) was first used in bladder perfusion therapy in the 1970s for the prevention and treatment of non-muscle-invasive bladder cancer (NMIBC). Intravesical instillation of BCG can significantly reduce cancer recurrence in NMIBC patients (71).

Despite being therapeutic targets in cancer treatment, bacteria can also regulate the effect of chemotherapy. *Escherichia coli* could influence the chemotherapy effect in gemcitabine and CB1954 by inducing drug resistance and activating cytotoxicity, separately (72, 73). As a cell taxon in pancreatic tumors, gammaproteobacteria can express an isoform of cytidine deaminase that inactivates gemcitabine, thereby reducing the concentration of gemcitabine in tumors and developing chemotherapy resistance (73). The number of *F. nucleatum* increased in CRC patients who relapsed after chemotherapy compared with those who did not relapse after chemotherapy (74). In CRC, *F. nucleatum* can biologically control chemotherapy resistance by coordinating TLR4-MyD88, miR18a and miR4802 and ULK1/ATG7 autophagy networks (74). This helps us to propose an important clinical question: are the conventional chemotherapy regimens including capecitabine plus oxaliplatin suitable for CRC patients with high expression of *F. nucleatum*? We suggest that chemotherapy regimens of patients with a high expression of *F. nucleatum* can be combined with anti-*F. nucleatum* treatment or autophagy inhibitors. A serious adverse reaction of irinotecan in cancer treatment is high intestinal toxicity. SN38, the active metabolite of irinotecan, is subsequently conjugated to SN38-G by hepatic UDP-glucuronyltransferase. SN38-G, an inactive metabolite of irinotecan, is excreted into the small intestine and then discharged from the body. The β-glucoronidases generated from intestinal bacteria can transform SN38-G into SN38, causing direct damage to the intestinal mucosa (75). Chemotherapy and associated mucosal damage may also affect the composition of the intestinal microbiome. Moreover, 5-fluorouracil (5-FU) can regulate the oral and fecal microbiome of laboratory animals by increasing Gram-negative anaerobic bacteria (76).

There was also an interaction between radiotherapy and ecological imbalance of intestinal flora. After radiotherapy, the imbalance of intestinal microbiota is characterized by the reduction of the abundance of symbiotic bifidobacteria, fecal bacilli, and *Clostridium* and the increase of *Bacteroides* and *Enterococcus* (77, 78). *In vitro* studies have shown that oral vancomycin induces the reduction of Gram-positive bacteria in the intestine and is associated with the enhanced efficacy of radiation therapy in melanoma, lung cancer, and cervical cancer models, which may possibly be through interferon-γ and CD8 T cell-dependent mechanisms (79). On the contrary, in the mouse model of breast cancer radiotherapy, antibiotics induced the decrease of *Clostridium* and the increase of intestinal yeast, promoting a macrophage-mediated tumor response (80). Intestinal bacteria may also be translocated through the damaged intestinal barrier, thereby regulating radiation toxicity, further contributing to uncontrolled intestinal immune response and tissue damage (81).

Compared with most other traditional drug delivery systems, bacteria have unique capabilities as drug carriers for cancer treatment. They can overcome physical barriers to target and accumulate in cancer tissues and initiate an anticancer immune response (82). The new discovery that bacteria exist in cancer cells (44) can promote the hypothesis that bacterial carriers can accurately deliver drugs to cancer cells.

In addition, bacteria can be genetically and chemically modified to produce and deliver anticancer agents to cancer tissues, thus improving the safety and effectiveness of cancer treatment while reducing the cytotoxicity to normal cells.

## Conclusions and prospects

In this review, we briefly summarize the recent progress in understanding the development or correlation between bacteria and cancers and the future research approaches beneficial to this field, including carrying drugs to help kill cancer cells. Visualization method showed that bacteria in cancers were primarily located in cancer and immune cells, rather than in the extracellular compartment (44, 45). The bacteria were found in close proximity to the nuclear membrane in human breast cancer tissues (44). The correlation between *F. nucleatum* and CRC has been extensively demonstrated. Targeting this bacterium in the gastrointestinal tract and the potential development of bacteria-targeted therapy against *F. nucleatum* are promising research approaches. FadA could specifically bind to the EC5 region of E-cadherin on CRC cells to attach to and invade CRC cells (58). Then, FadA can activate the β-catenin signaling to promote CRC cell proliferation (58). *F. nucleatum* can also invade ESCC cancer cells and promote cancer progression through the NOD1/RIPK2/NFκB pathway. This is a specific study of how bacteria entered tumor cells and played their roles (**Figure 1**).

Studying the causal relationship and molecular interaction between bacteria and cancers promises to provide new clues for the development, progression, and treatment response of human cancers. In addition to trying to understand bacterial associations and causality in cancer, such research also faces arduous challenges related to sample allocation, processing, sequencing, and data analysis. Despite these challenges, the contribution of bacteria to cancer biology may occupy a central position in cancer research over the next decade, making more contributions to cancer diagnosis, patient stratification, and treatment.

## Limitation

However, the way other bacteria enter cancer cells and how diseases affect tumor progression in which way still require further study. There are still many questions that need to be explored. What is the origin of bacteria in cancer? Does the composition of the bacteria change with cancer progression? Do bacteria “travel” with cancer cells to metastatic sites? Are there any bacteria at metastasis sites more relevant to the new location? We should focus on exploring and solving the above problems to better contribute to the treatment of cancers.

## Author contributions

WZ and JW investigated and wrote the original draft. ZL and JW proposed the ideas and edited the manuscript. All authors contributed to the article and approved the submitted version.

## Funding

This work was supported by Natural Science Foundation of Jiangsu Province (BK20201090), China Postdoctoral Science Foundation (2021M691338), and Young Scholars Fostering Fund of the First Affiliated Hospital of Nanjing Medical University (PY2021052).

## Conflict of interest

The authors declare that the research was conducted in the absence of any commercial or financial relationships that could be construed as a potential conflict of interest.

## Publisher’s note

All claims expressed in this article are solely those of the authors and do not necessarily represent those of their affiliated organizations, or those of the publisher, the editors and the reviewers. Any product that may be evaluated in this article, or claim that may be made by its manufacturer, is not guaranteed or endorsed by the publisher.
